# Topogami: Topologically Linked DNA Origami

**DOI:** 10.1021/acsnanoscienceau.1c00027

**Published:** 2021-11-12

**Authors:** Yusuke Sakai, Gerrit D. Wilkens, Karol Wolski, Szczepan Zapotoczny, Jonathan G. Heddle

**Affiliations:** † Bionanoscience and Biochemistry Laboratory, Malopolska Centre of Biotechnology, Jagiellonian University, Gronostajowa 7A, 30-387 Krakow, Poland; ‡ Postgraduate School of Molecular Medicine, Żwirki I Wigury 61, 02-091 Warsaw, Poland; § Faculty of Chemistry, Jagiellonian University, Gronostajowa 2, 30-387 Krakow, Poland

**Keywords:** DNA origami, Tn3 resolvase, catenane, topological links, topoisomerase, DNA nanotechnology, molecular machine, AFM

## Abstract

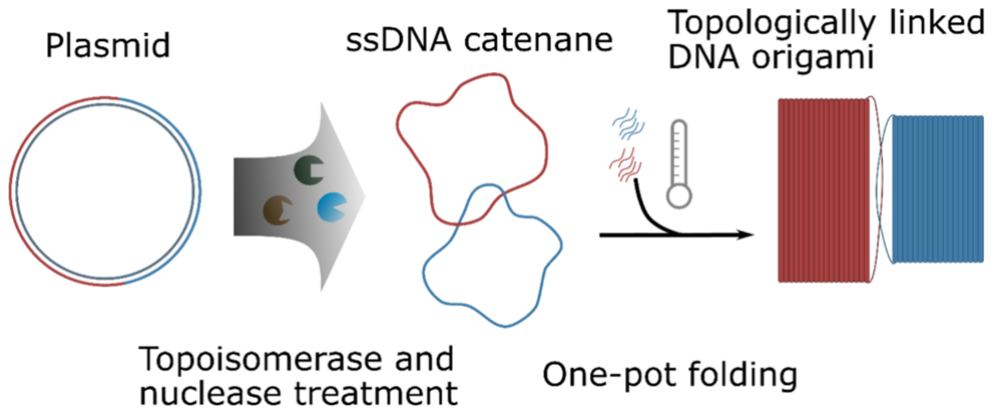

DNA origami is a widely used DNA nanotechnology that allows construction of two-dimensional and three-dimensional nanometric shapes. The designability and rigidity of DNA origami make it an ideal material for construction of topologically linked molecules such as catenanes, which are attractive for their potential as motors and molecular machines. However, a general method for production of topologically linked DNA origami has been lacking. Here, we show that catenated single-stranded DNA circles can be produced and used as a universal scaffold for the production of topologically linked (catenated) DNA origami structures where the individual linked structures can be of any arbitrary design. Assembly of these topologically linked DNA origami structures is achieved via a simple one-pot annealing protocol.

## Introduction

Topologically linked molecules such as catenanes and rotaxanes are challenging and fascinating targets in supramolecular chemistry.^1^ They achieved particular prominence after Sauvage’s demonstration of the templated approach to molecular catenane synthesis in 1983^2,3^ with Stoddart demonstrating a rotaxane almost a decade later.^4^ Since then, there have been many examples of topological molecules,^1^ and demonstrated applications include nanometric electronic switches, liquid crystals, and other new materials.^5^

DNA origami is a DNA nanotechnology in which a long single-stranded DNA “scaffold” is shaped by the action of many short “staple” strands, which bind to cognate sequences distributed throughout the scaffold.^6,7^ Typically, the scaffold strand is thousands of bases long, providing enough material for the construction of complex, rigid structures such as dynamic containers whose opening can be programmed in response to stimuli.^8,9^ While customization of scaffolds can be challenging, numerous examples have been reported, including those that are shortened,^10,11^ extended,^12−14^ or otherwise modified to provide arbitrary length and sequences.^15−18^ Efforts to connect together discrete DNA origami structures have included hybridization of sticky ends^19^ or base stacking^7,20−22^ and have achieved gigadalton-scale structures^23^ and fully addressable semimicrometer-scale tiles.^24^

Topologically linked DNA molecules are known to occur in nature^25^ and are also attractive as artificial constructs in DNA nanotechnology. In recent years, catenated ssDNA rings have been designed and produced in vitro using a number of methods. Typically, they involve enzymatic ligation or chemical coupling of short linear DNA components following geometric prearrangement by DNA origami,^26^ hybridization of short complementary sequences^27−30^ or conjugation with dsDNA-binding moieties.^31^ The resulting structures have been shown capable of functioning as switches^27,32^ and rotary motors.^28,33^ However, this approach results in small (sub-200 nt) catenanes, essentially an order of magnitude smaller than required to make complex DNA origami structures. Production of considerably longer catenated ssDNA circles of a length suitable for DNA origami has been demonstrated in a study, which used Ena/Vasp-like protein and topoisomerase I.^34^ However, this approach showed poor efficiency, generated a range of catenated products, and has not been applied for DNA origami production.

Despite these challenges, topologically linked DNA origami remains an attractive goal due to its high molecular weight and resulting greater structural redundancy compared to classical topologically linked molecules. As a result, it has the potential to construct molecular machines with increased functionality and sophistication due to the ability to irreversibly link together discrete DNA origami structures.

Very few examples of topologically linked DNA origami structures have been demonstrated to date. The first that we are aware of was in 2010 when a two-ring DNA origami catenane was produced.^35^ This was achieved by splitting a DNA origami Mobius strip followed by removal of selected staple strands. In this case, the topology of the two connected scaffold strands is such that decatenation can be achieved without covalent bond breaking. More recently, a second two-ring DNA origami catenane was demonstrated. Here, each of the two rings was made from four individual DNA origami structures, which were joined together via additional staple strands with the help of a gold particle templated approach.^36^ As with the first example, removal of noncovalently bound staple strands results in decatenation of the structure. In contrast, a truly catenated scaffold can only be decatenated by covalent bond breaking and may be preferable for the extra stability it would provide to the linked DNA origami components.

Two-component DNA origami structures, which could benefit from being produced as true catenanes, include rotaxanes, the first of which was demonstrated in 2016^37^ in a structure where two discrete DNA origami structures constituted the axle and ring. These were assembled first with the ring in an open form, which was then partially wrapped around the axle before being mechanically “clamped” in place by complementary sequences extended from staple strands. More recently, another rotaxane was produced from a single template DNA origami structure wherein the ring and the axle of the rotaxane precursor were connected via ssDNA regions, which were then specifically removed by Cas12a to produce the final rotaxane.^38^

A common pattern in all topologically linked DNA origami structures to date is that they are specifically constructed such that predetermined DNA origami subunits are positioned with respect to each other. This is followed by disconnection of the “linkers” (a subset of staples, base pairing at specific sites, and a part of the scaffold) resulting in a topologically linked product. A consequence of this approach is that the construction method is design specific. The final, folded DNA origami structures are required to be produced first, prior to the topological linkage being formed. This is in contrast to the original DNA origami concept whereby starting with a scaffold strand, almost any arbitrary structure can be designed and produced.

In this work, we aimed to produce a topologically linked two-component DNA origami structure where the linkage is made at the level of two single-stranded scaffold strands. To do this, we developed a method for preparation of a pair of topologically interlocked circular ssDNA scaffold rings using Tn3 resolvase. Catenating at this fundamental level has two noteworthy outcomes shown here for the first time. First, the system is universal: as catenation does not depend on the formation of a specific DNA origami structure, the catenated ssDNA rings we produced can, in principle, be used to construct almost any two origami structures in a catenated form. Second, unlike topologically linked DNA origami structures to date, the topological linkage can only be undone by breaking a covalent bond (i.e., DNA backbone cleavage), resulting in high stability of the produced structures.

## Results

### Production of Catenated ssDNA Scaffold Strands

**Figure 1:**
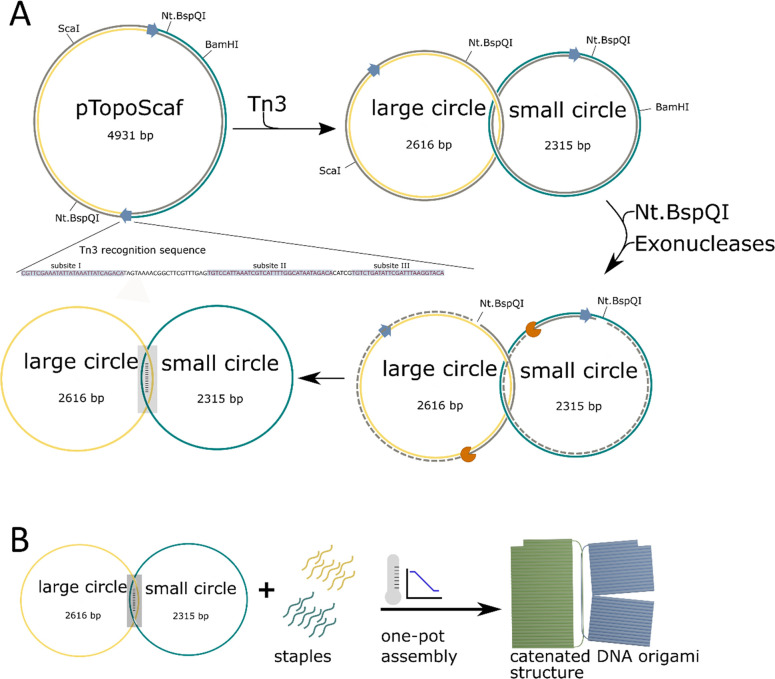
Overview of the catenane scaffold production process by a combination of DNA recombination nicking and exonuclease digestion. (a) First, supercoiled plasmid with two parallel recombination sites is treated with Tn3 resolvase, leading to the production of catenane dsDNA circles. Next, both circles are nicked at one specific site on each circle on opposite strands of the original plasmid. One strand of each double stranded circle is removed by exonuclease treatment, generating a single-stranded catenane with a 250 nt complementary region between both circles. (b) Single-stranded catenanes can be used directly as a scaffold source for one-pot assembly of covalently connected DNA origami structures linked by a scaffold loop.

We designed pTopoScaf, a vector consisting of two scaffold domains connected by parallel recognition sequences (*res*) in a negatively supercoiled parent plasmid,^39^ which are processed by Tn3 resolvase, a serine recombinase,^40−42^ as outlined in Figure 1. The enzyme catalyzes a site-specific recombination of negatively supercoiled circular DNA at the *res* sites to form a supercoiled catenane,^43^ while the reverse reaction occurs in the case of the relaxed substrate.^39^ The recognition sequence consists of three domains (sites I–III): site I is a cleavage site, and sites II–III are required for recombination^40^ (Figure 1).

**Figure 2:**
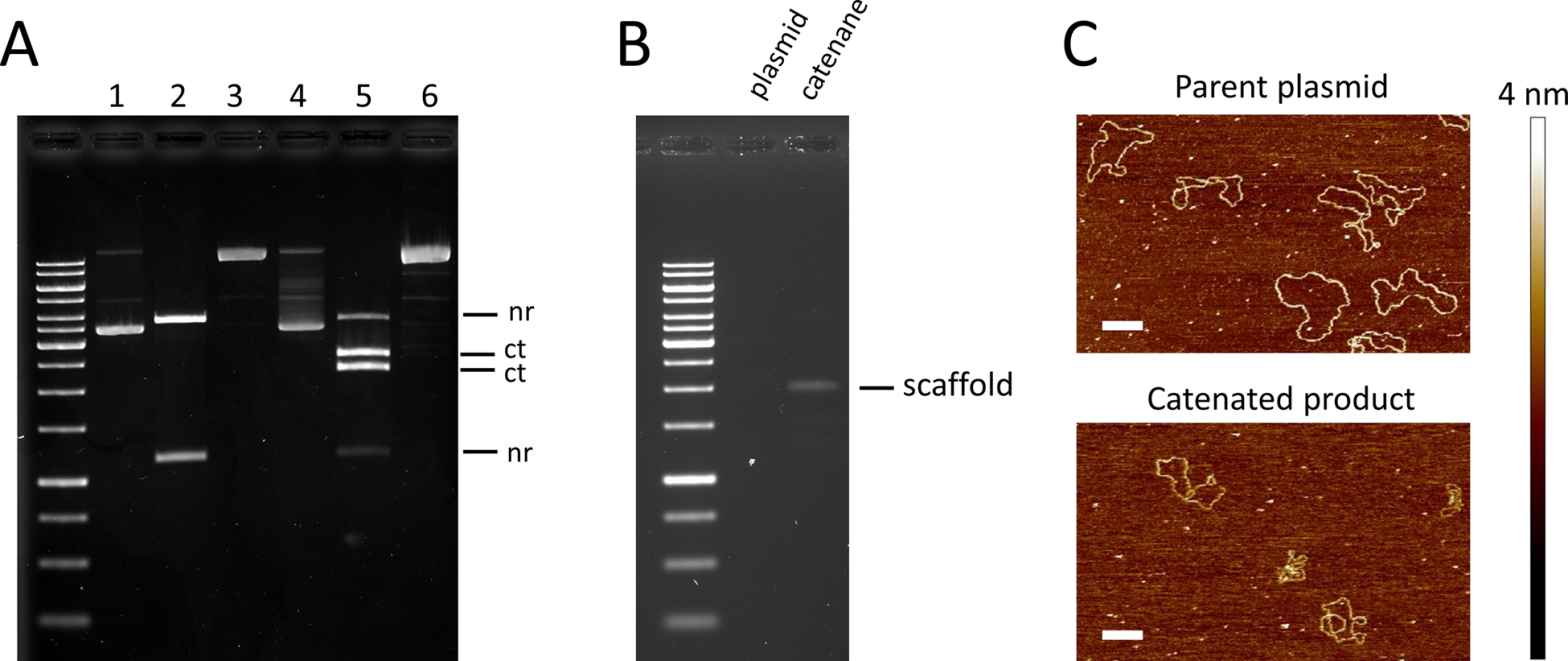
Preparation of the catenated scaffold from the parent plasmid. (a) Catenation of circular dsDNA using Tn3 recombinase. Agarose gel image of (1) undigested parent plasmid, (2) plasmid nicked with *Bam*HI and *Sca*I, (3) plasmid nicked with Nt.BspQI, (4) undigested catenane after Tn3 catenation, (5) catenane digested with *Bam*HI and *Sca*I showing appearance of digestion products from catenane rings (2.3 and 2.6 kbp products), and (6) catenane nicked with Nt.BspQI. (b) Exonuclease digestion of nicked plasmid and catenane. Formation of ssDNA catenane is indicated by a single band showing a higher electrophoretic mobility compared to the untreated catenane. The exonuclease-treated plasmid appears to be degraded, indicating efficient removal of noncatenane plasmid from the catenane sample after exonuclease digest. (c) AFM micrograph of the plasmid substrate (top, pMA21) and its catenane (bottom) after treatment with Nt.BspQI and Nb.BsmI nickase to remove negative supercoiling. Note that the sequences of those DNA strands differed in one nucleotide from pTopoScaf and its catenane product shown in the agarose image. Scale bars are 300 nm. Ladder in panels (a) and (b): GeneRuler 1 kb (Thermo Fisher Scientific). Bands correspond (from top to bottom) to 20,000; 8000; 6000; 5000; 4000; 3500; 3000; 2500; 2000; 1500; 1000; 750; 500; and 250 bp.

We integrated Nt.BspQI nickase sites on opposite strands of the parent vector such that after Tn3 mediated recombination, each ring of the two-ring catenane would bear a single nickase site. This allows conversion of the catenated product into ssDNA by Nt.BspQI nicking and subsequent exonuclease digestion, while the nicked noncatenated parent plasmid will be removed by exonuclease digestion (Figure 2B). Note that due to complementary sequences at the *res* sites of the two catenated strands, they hybridize to each other forming a 250 bp dsDNA domain, including linker sequences, though this does not change the topology of the catenated product.

Production of catenated scaffolds was confirmed by agarose gel electrophoresis where *Sca*I/*Bam*HI double digest of the Tn3-treated plasmid indicated the recombination event leading to formation of two smaller circles, each bearing one cutting site (for the relative position of *Bam*HI and *Sca*I cutting sites in the plasmid and catenane, please refer to Figure 1). The undigested plasmid and catenane were visible as a major band running at the same height, confirming that the catenane circles were indeed covalently connected (Figure 2A). This was supported by the consistent pattern of *Bam*HI/*Sca*I double-digest products of each (Figure 2A).

Further confirmation was provided by atomic force microscopy (AFM) analysis, which showed clear evidence of the catenated product compared to unreacted controls (Figure 2C). Catenated samples were subsequently treated with Nt.BspQI, leading to one major band consisting of catenanes in which each circle in the catenane was nicked once, while the unreacted parent plasmid contained two nicks, one in each strand. As expected, the nicked catenane sample appeared as a single major band on the gel, running higher than the catenane and plasmid samples. Next, the plasmid was digested using exonuclease III and exonuclease I, removing the parent plasmid while leaving one strand of each of the catenane circles intact (Figure 2B). The purity of the catenated scaffold pair was shown as a clear single major band on the agarose gel, while faint smearing over the band might imply the contamination of byproducts.

### Production of Catenated DNA Origami

**Figure 3:**
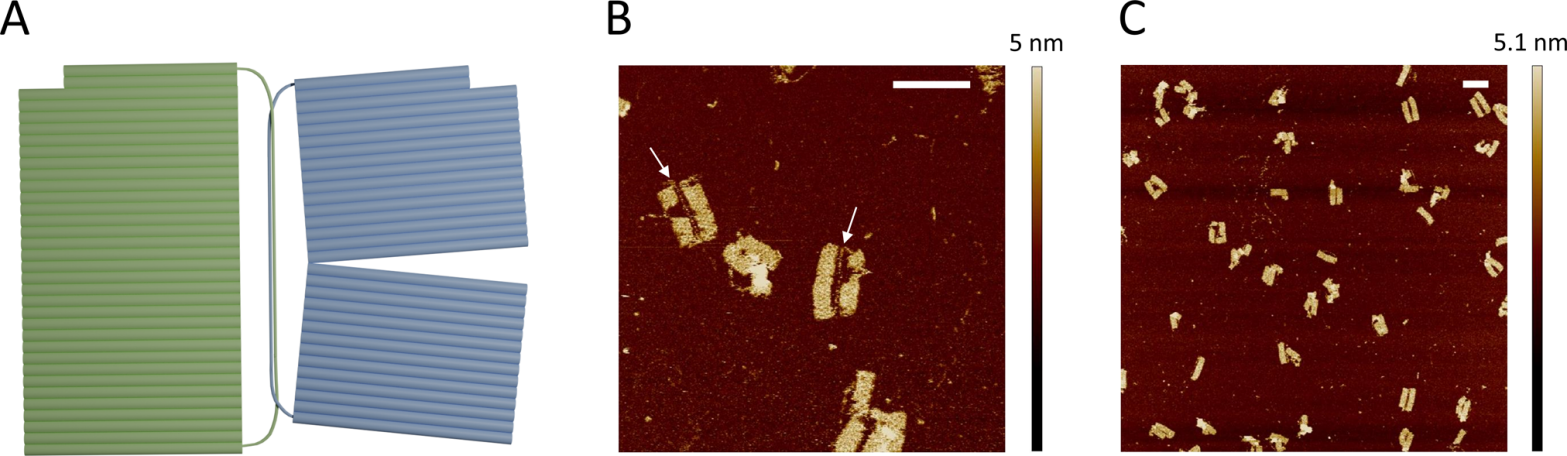
Topogami structure assembly from the catenated scaffold. (A) Schematic shape of designed topogami: two different sizes of rectangles interlocked by dsDNA loops. (B) Typical AFM image of the assembled topogami structure. White arrows indicate 250 bp dsDNA loops. Scale bars are 100 nm. (C) A wider field, typical AFM image of the assembled topogami structures. Note that the catenated origami structures are free to rotate relative to each other, meaning that the smaller structure may open toward or away from the larger structure. Scale bars are 100 nm.

**Figure 4:**
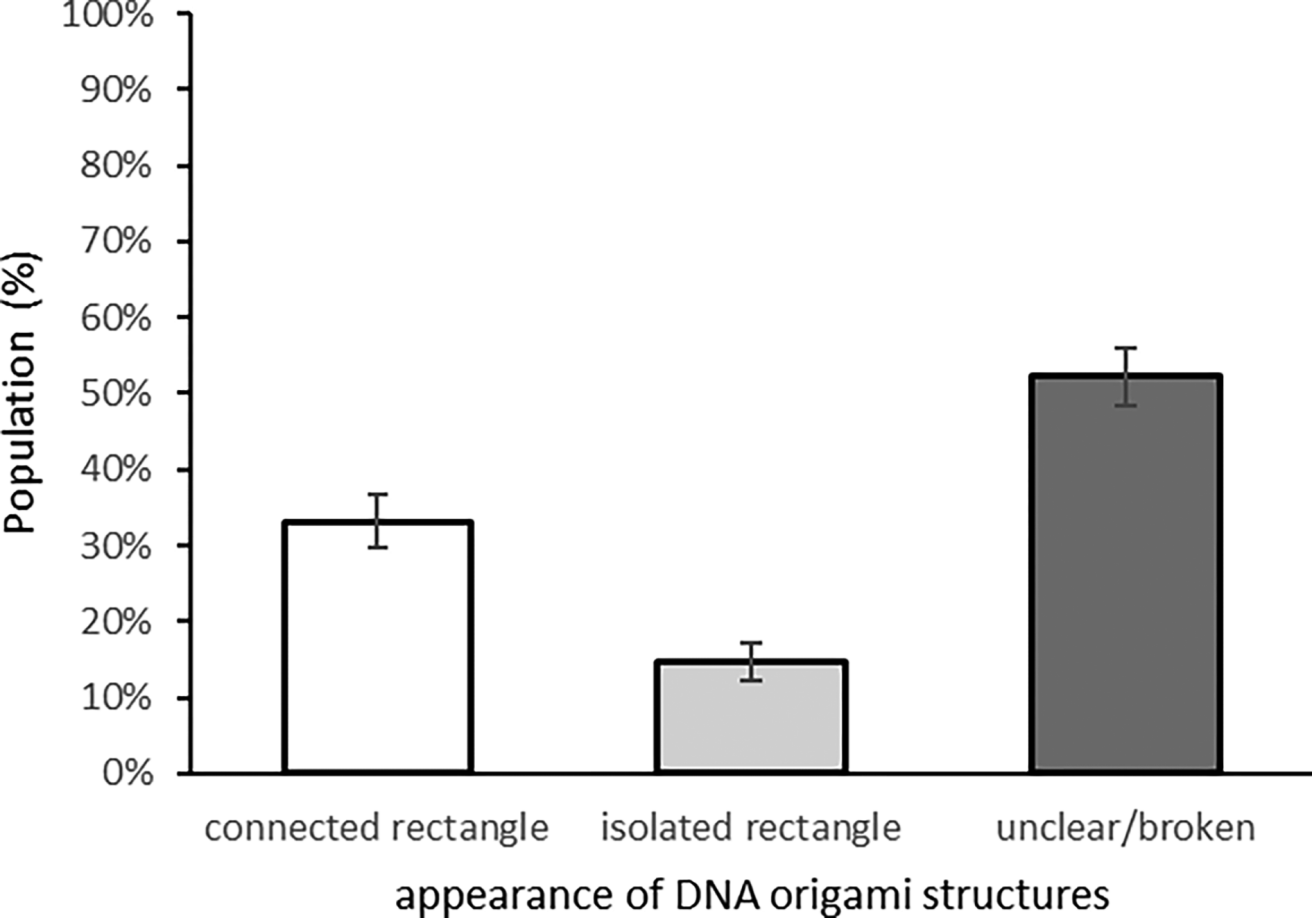
Analysis of formed DNA origami catenane structures. About 50% of structures clearly showed either catenated pairs or isolated structures (*n*: 184).

Using the catenated scaffold, we designed a topologically interlocked DNA origami complex, which we named “topogami.” Topogami consists of two discrete DNA origami structures, which are catenated at the level of the scaffold strands. To demonstrate this, we chose two rectangular single-layer DNA origami structures as the two linked structures (Figure 3A) wherein the smaller rectangle was partially opened in its central region by omission of staple strands, making this structure easier to distinguish in imaging. The formation of the topogami structure was achieved by a typical DNA origami annealing reaction and then analyzed by AFM. This showed the presence of an as-designed dimeric structure consisting of two interconnected rectangles with dimensions of 90 × 24 nm and 80 × 24 nm, respectively. By definition, the two rings of the topogami must be interlocked. This could prove disruptive if the point of interlocking interferes with the folding of the origami. In addition, our prototype topogami design contains 250 complementary bases on each of the two ssDNA rings, which could also have disruptive effects if they competed with staple strand binding. Both of these challenges were overcome in a single solution where the complementary sequence was kept discrete from the folded origami. To achieve this, a double helix domain utilizing the existing 250 nt complementary sequence was designed to be located between the two connected DNA origami structures and was successfully observed in AFM and high-speed AFM imaging (Figure 3B and 3C). Statistical analysis of the AFM image revealed that when considering folded structures, only the majority (69% ± 5%) of the assembled products were catenated. Clearly isolated (decatenated) “half” structures were also observed with a substantially lower frequency (31% ± 5%) (Figures 4 and S3 and Table S1). Note that only clearly paired rectangles and obviously isolated rectangles were counted to minimize bias (total 88 particles) and the others were separately classified as unclear particles (Figure S3 and Table S3). This could lead to an underestimate of yield of the correct product in cases where it was not obvious. Interestingly, in some correctly catenated structures, the small rectangle did not appear in the open form possibly due to restrictive effects exerted by the linked large rectangle.

Overall, the data clearly showed the successful demonstration of topologically linked DNA origami used to produce two discrete and covalently catenated DNA origami structures.

## Discussion

The original DNA origami concept provided a single scaffold strand, which, by the addition of staple strands, could fold into any arbitrary shape. Analogously, the DNA topogami approach provides a universal template for producing topologically linked DNA origami. In principle, this can allow construction of any arbitrary catenated DNA origami structures containing more than one DNA origami structure by simple annealing of a mixture of template strands and staples.

One unique outcome of the topogami approach is in the irreversibility of the topological linkage produced between the two template strands. All topologically linked DNA origami structures produced to date are only able to form the topological linkage by a “gap-closing” reaction whereby one DNA origami structure “wraps around” the other through the action of staple strands, i.e., by using base pairing. In our case, a true catenane is produced, as the template strands themselves are catenated, meaning that decatenation would require cleavage of a covalent bond in the DNA backbone.

While topogami production was achieved, some separated components were observed: this may be due to (i) an incomplete exonuclease reaction leaving one scaffold as double stranded, resulting in an ability to anneal with staple strands; (ii) nicking of the scaffold during high-temperature annealing or storage, allowing two halves of the topogami to separate; and (iii) dissociation of one rectangle due to interaction with the cantilever probe during scanning.

In light of this first demonstration of the topogami concept, we have identified a number of avenues for future development. This includes (i) increasing the number of catenated scaffold strands beyond two. This would allow larger, stably linked DNA origami structures to be formed; (ii) shortening the complementary loop sequence between interlinked origami structures to 28 bp by utilizing a Tn3 resolvase mutant (D102Y), which is capable of catalyzing a parallel pair of *res* sites even when one of them lacks sites II and III^40^ though with a slightly lower efficiency; (iii) entire removal of the complementary *res* sequence by adding an extra site-specific recombination step using Flp or Cre;^44^ and (iv) alternatively the complementary sequences could be denatured and utilized as standard scaffold strands.^45^

We suggest that topogami may prove useful for producing functional DNA origami systems that benefit from being irreversibly linked. For example, given the proven utility of catenanes and related structures such as rotaxanes as motors and switches along with the high programmability of DNA origami, we expect that this proof of principle work will allow the design and construction of more complex and functional, topologically linked DNA origami systems.

## Conclusions

In summary, we have demonstrated a novel method for the construction of single-stranded DNA catenanes at the kb length scale. The catenanes can be used for the assembly of covalently connected DNA origami scaffolds. DNA origami is well-established as a flexible method for production of functional nanometric objects. It has the attractive feature that a multitude of structures can be produced using a universal approach based on an ss scaffold DNA. Concurrently, work with small, covalently catenated circular DNA structures has highlighted their stability and possibility for use as molecular motors. In this work, we have combined both approaches, producing large, catenated ssDNA circles, which can be used as “universal” scaffolds to produce covalently catenated DNA origami (“topogami).” The ability to topologically link discrete origami modules in this way may lead to increased functionality and more stable DNA origami-based machines.

## Materials and Methods

Plasmid pMA21^46^ containing two of parallel Tn3 *res* sites and *Escherichia coli* DH5 (*F*^–^ × λ^–^ Δ(lacZYA-argF)U169 recA1 endA1 hsdR17 (r_K_^–^, m_K_^+^) phoA supE44 thi*-*1 gyrA96 relA1) amplification host cells were a gift from Prof. Marshall Stark.

### Plasmid Mutagenesis

Plasmid pTopoScaf was derived from pMA21 by introducing a second Nt.BspQI nickase site (GCTCTTCN↓) by polymerase chain reaction (PCR) using the following mutagenic primers:

Nt.BspQI_for 5’GATAAGCTGTCAAAGCTCTTCATGAGAATTCGC3’ and Nt.BspQI_rev 5’AATTCTCATGAAGAGCTTTGACAGCTTATCATCG3’.

Amplification was performed in a 20 μL reaction in 1× HF buffer (NEB), containing 50 ng of template DNA, 1 μM concentration of each primer, 500 μM dNTP mix, and 1 μL of Phusion polymerase. The PCR cycling program was at 98 °C for 60 s followed by 20 cycles at 98 °C for 15 s; 55 °C for 15 s, and 72 °C for 140 s and a final elongation step at 72 °C for 4 min. After completing the PCR reaction, methylated template DNA was removed by incubation with 1 μL of FastDigest *Dpn*I (Thermo Fisher Scientific) for 15 min at 37 °C. An aliquot of 5 μL was transformed into chemo-competent *E. coli* DH5 and plated on Luria-Bertani plates containing 100 μg/mL ampicillin. The resulting mutant plasmid was verified by DNA sequencing.

### Plasmid Mutagenesis

Supercoiled plasmid DNA of pTopoScaf was purified from transformed *E. coli* strain DH5. Bacteria were grown over night in LB containing 100 μg/mL ampicillin. Plasmid was isolated using either the GenElute HP Select Plasmid Gigaprep Kit (Sigma Aldrich) or GeneJET Plasmid Miniprep Kit (Thermo Fisher Scientific). DNA concentration was estimated from absorbance at 260 nm using a Nanodrop ND-1000 spectrophotometer. If required, DNA was concentrated using EtOH precipitation.

### Preparation of the Scaffold Catenane

Tn3 resolvase was a kind gift of Prof. Marshall Stark. Tn3 resolvase was diluted and stored in storage buffer (20 mM Tris–HCl (pH 7.5), 1 mM DTT, 0.1 mM EDTA, 1 M NaCl, and 50% v/v glycerol). Plasmid catenation was carried out as previously reported^42^ with slight modifications. In brief, Tn3 resolvase was diluted 20-fold into 50 μL of reaction buffer (10 mM MgCl_2_, 0.1 mM EDTA, and 50 mM Tris–HCl, pH 8.2) containing 2.5 μg of DNA. Reactions were carried out for 2 h at 37 °C. The Tn3 catenation reaction was stopped by heating for 10 min at 70 °C. After cooling to room temperature, 2 μL of Nt.BspQI (NEB, 10,000 U/mL) was added and the reaction was incubated for 1 h at 50 °C followed by 20 min at 80 °C. The nicked DNA was diluted with 50 μL of 1× Exo III buffer (6.6 mM Tris–HCL pH 8 and 0.66 mM MgCl_2_). Then, 1 μL of exonuclease III (Thermo Fisher Scientific, 200 U/μL) and 4 μL of exonuclease I (Thermo Fisher Scientific, 10 U/μL) were added and incubated for 4 h at 37 °C followed by 10 min at 70 °C. Incubations were carried out in 50 μL aliquots in a thermocycler with a heated lid. Reactions were analyzed by running 100 ng of DNA on a 1.2% agarose gel followed by subsequent staining with ethidium bromide and imaging using a UV transilluminator. To purify single-stranded DNA from enzymes, four starting reactions were pooled purified with phenol/chloroform extraction and concentrated 10 times by EtOH precipitation and resuspension in milli-Q water.

### DNA Origami Preparation

For design of DNA origami structures cadnano2 was used.^47^ DNA origami structures were assembled in a one-pot reaction by mixing the catenated scaffold and staple strands at final concentrations of 10 and 60 nM, respectively. Folding buffer contained 5 mM Tris, 1 mM EDTA, and 10 mM MgCl_2_. To assemble structures, DNA strands were incubated at 80 °C for 10 min followed by cooling from 79 to 25 °C with a decrease of 1 °C per 1 min in a thermocycler.

### AFM Analysis

First, 1.5 μL of 2 nM assembled DNA origami sample was applied to freshly cleaved mica and incubated for 1 min followed by addition of 20 μL of folding buffer and immediately by 1.5 μL of 100 mM NiCl_2_. The specimen was measured in liquid by AFM (Dimension Icon, Bruker) working in PeakForce QNM mode. ScanAsyst-Fluid+ probes (Bruker) with a nominal spring constant equal to 0.7 N/m and a sharpened tip necessary for high-resolution imaging in fluid (nominal radius equal to 2 nm) were used in all the measurements. Figure 3B,C and Figure S3 were obtained using MutiMode-8 AFM (Bruker) using a BL-AC40TS-C2 probe with the same method.

HS-AFM analysis was performed using a bespoke HS-AFM (NanoLSI, Kanazawa University) with a BL-AC10FS-A2 cantilever probe. The DNA origami sample was loaded on freshly cleaved and nickel chloride-treated mica and imaged in folding buffer in tapping mode in liquid.

## Supporting Information Available

Optimization of plasmid amplification; amplification of pMA21 in *E. coli* Dh5 alpha; cadnano diagram of rectangle catenanes; scaffold sequences; staple list for large rectangles; statistical analysis of AFM images; summary of AFM image analysis; and cadnano diagram of the topogami rectangle and “T” shape catenanes; staple list for “T” shape DNA origami (PDF)

## References

[ref1] Gil-Ramírez G.; Leigh D. A.; Stephens A. J. Catenanes: fifty years of molecular links. Angew Chem Int Ed Engl 2015, 54, 6110–6150. 10.1002/anie.201411619. 25951013 PMC4515087

[ref2] Dietrich-Buchecker C. O.; Sauvage J. P.; Kintzinger J. P. Une nouvelle famille de molecules : les metallo-catenanes. Tetrahedron Lett. 1983, 24, 5095–5098. 10.1016/S0040-4039(00)94050-4.

[ref3] Dietrich-Buchecker C. O.; Sauvage J. P.; Kern J. M. Templated synthesis of interlocked macrocyclic ligands: the catenands. J. Am. Chem. Soc. 1984, 106, 3043–3045. 10.1021/ja00322a055.

[ref4] Bissell R. A.; Córdova E.; Kaifer A. E.; Stoddart J. F. A chemically and electrochemically switchable molecular shuttle. Nature 1994, 369, 133–137. 10.1038/369133a0.

[ref5] Moulin E.; Faour L.; Carmona-Vargas C. C.; Giuseppone N. From Molecular Machines to Stimuli-Responsive Materials. Adv. Mater. 2020, 32, e1906036 10.1002/adma.201906036. 31833132

[ref6] Wagenbauer K. F.; Engelhardt F. A. S.; Stahl E.; Hechtl V. K.; Stömmer P.; Seebacher F.; Meregalli L.; Ketterer P.; Gerling T.; Dietz H. How We Make DNA Origami. ChemBioChem 2017, 18, 1873–1885. 10.1002/cbic.201700377. 28714559

[ref7] Rothemund P. W. Folding DNA to create nanoscale shapes and patterns. Nature 2006, 440, 297–302. 10.1038/nature04586. 16541064

[ref8] Douglas S. M.; Bachelet I.; Church G. M. A logic-gated nanorobot for targeted transport of molecular payloads. Science 2012, 335, 831–834. 10.1126/science.1214081. 22344439

[ref9] Tang M. S. L.; Shiu S. C.; Godonoga M.; Cheung Y. W.; Liang S.; Dirkzwager R. M.; Kinghorn A. B.; Fraser L. A.; Heddle J. G.; Tanner J. A. An aptamer-enabled DNA nanobox for protein sensing. Nanomedicine 2018, 14, 1161–1168. 10.1016/j.nano.2018.01.018. 29410111

[ref10] Brown S.; Majikes J.; Martínez A.; Girón T. M.; Fennell H.; Samano E. C.; Labean T. H. An easy-to-prepare mini-scaffold for DNA origami. Nanoscale 2015, 7, 16621–16624. 10.1039/C5NR04921K. 26413973

[ref11] Said H.; Schüller V. J.; Eber F. J.; Wege C.; Liedl T.; Richert C. M1.3 – a small scaffold for DNA origami. Nanoscale 2013, 5, 284–290. 10.1039/C2NR32393A. 23160434

[ref12] Marchi A. N.; Saaem I.; Vogen B. N.; Brown S.; LaBean T. H. Toward larger DNA origami. Nano Lett. 2014, 14, 5740–5747. 10.1021/nl502626s. 25179827

[ref13] Krieg E.; Shih W. M. Selective Nascent Polymer Catch-and-Release Enables Scalable Isolation of Multi-Kilobase Single-Stranded DNA. Angew Chem Int Ed Engl 2018, 57, 714–718. 10.1002/anie.201710469. 29210156

[ref14] Zhang H.; Chao J.; Pan D.; Liu H.; Huang Q.; Fan C. Folding super-sized DNA origami with scaffold strands from long-range PCR. Chem. Commun. 2012, 48, 6405 10.1039/c2cc32204h. 22618197

[ref15] Erkelenz M.; Bauer D. M.; Meyer R.; Gatsogiannis C.; Raunser S.; Saccà B.; Niemeyer C. M. A Facile Method for Preparation of Tailored Scaffolds for DNA-Origami. Small 2014, 10, 73–77. 10.1002/smll.201300701. 23861344

[ref16] Zadegan R. M.; Jepsen M. D.; Thomsen K. E.; Okholm A. H.; Schaffert D. H.; Andersen E. S.; Birkedal V.; Kjems J. Construction of a 4 zeptoliters switchable 3D DNA box origami. ACS Nano 2012, 6, 10050–10053. 10.1021/nn303767b. 23030709

[ref17] Engelhardt F. A. S.; Praetorius F.; Wachauf C. H.; Brüggenthies G.; Kohler F.; Kick B.; Kadletz K. L.; Pham P. N.; Behler K. L.; Gerling T.; Dietz H. Custom-Size, Functional, and Durable DNA Origami with Design-Specific Scaffolds. ACS Nano 2019, 13, 5015–5027. 10.1021/acsnano.9b01025. 30990672 PMC6992424

[ref18] Nafisi P. M.; Aksel T.; Douglas S. M. Construction of a novel phagemid to produce custom DNA origami scaffolds. Synth Biol (Oxf) 2018, 3, ysy015 10.1093/synbio/ysy015. 30984875 PMC6461039

[ref19] Liu W.; Zhong H.; Wang R.; Seeman N. C. Crystalline Two-Dimensional DNA-Origami Arrays. Angewandte Chemie International Edition 2011, 50, 264–267. 10.1002/anie.201005911. 21053236 PMC3463376

[ref20] Douglas S. M.; Dietz H.; Liedl T.; Högberg B.; Graf F.; Shih W. M. Self-assembly of DNA into nanoscale three-dimensional shapes. Nature 2009, 459, 414–418. 10.1038/nature08016. 19458720 PMC2688462

[ref21] Gerling T.; Wagenbauer K. F.; Neuner A. M.; Dietz H. Dynamic DNA devices and assemblies formed by shape-complementary, non-base pairing 3D components. Science 2015, 347, 1446–1452. 10.1126/science.aaa5372. 25814577

[ref22] Woo S.; Rothemund P. W. Programmable molecular recognition based on the geometry of DNA nanostructures. Nat. Chem. 2011, 3, 620–627. 10.1038/nchem.1070. 21778982

[ref23] Wagenbauer K. F.; Sigl C.; Dietz H. Gigadalton-scale shape-programmable DNA assemblies. Nature 2017, 552, 78–83. 10.1038/nature24651. 29219966

[ref24] Tikhomirov G.; Petersen P.; Qian L. Fractal assembly of micrometre-scale DNA origami arrays with arbitrary patterns. Nature 2017, 552, 67–71. 10.1038/nature24655. 29219965

[ref25] Martinez-Robles M. L.; Witz G.; Hernandez P.; Schvartzman J. B.; Stasiak A.; Krimer D. B. Interplay of DNA supercoiling and catenation during the segregation of sister duplexes. Nucleic Acids Res. 2009, 37, 5126–5137. 10.1093/nar/gkp530. 19553196 PMC2731910

[ref26] Cassinelli V.; Oberleitner B.; Sobotta J.; Nickels P.; Grossi G.; Kempter S.; Frischmuth T.; Liedl T.; Manetto A. One-Step Formation of “Chain-Armor”-Stabilized DNA Nanostructures. Angew Chem Int Ed Engl 2015, 54, 7795–7798. 10.1002/anie.201500561. 25980669

[ref27] Elbaz J.; Wang Z. G.; Wang F.; Willner I. Programmed dynamic topologies in DNA catenanes. Angew Chem Int Ed Engl 2012, 51, 2349–2353. 10.1002/anie.201107591. 22287100

[ref28] Lu C.-H.; Cecconello A.; Elbaz J.; Credi A.; Willner I. A Three-Station DNA Catenane Rotary Motor with Controlled Directionality. Nano Lett. 2013, 13, 2303–2308. 10.1021/nl401010e. 23557381

[ref29] Liu D.; Chen G.; Akhter U.; Cronin T. M.; Weizmann Y. Creating complex molecular topologies by configuring DNA four-way junctions. Nat. Chem. 2016, 8, 907–914. 10.1038/nchem.2564. 27657865

[ref30] Lohmann F.; Valero J.; Famulok M. A novel family of structurally stable double stranded DNA catenanes. Chem. Commun. 2014, 50, 6091–6093. 10.1039/C4CC02030H. 24777123

[ref31] Schmidt T. L.; Heckel A. Construction of a Structurally Defined Double-Stranded DNA Catenane. Nano Lett. 2011, 11, 1739–1742. 10.1021/nl200303m. 21410245

[ref32] Qi X.-J.; Lu C.-H.; Liu X.; Shimron S.; Yang H.-H.; Willner I. Autonomous Control of Interfacial Electron Transfer and the Activation of DNA Machines by an Oscillatory pH System. Nano Lett. 2013, 13, 4920–4924. 10.1021/nl402873y. 23988015

[ref33] Valero J.; Pal N.; Dhakal S.; Walter N. G.; Famulok M. A bio-hybrid DNA rotor-stator nanoengine that moves along predefined tracks. Nat. Nanotechnol. 2018, 13, 496–503. 10.1038/s41565-018-0109-z. 29632399 PMC5994166

[ref34] Takaku M.; Takahashi D.; Machida S.; Ueno H.; Hosoya N.; Ikawa S.; Miyagawa K.; Shibata T.; Kurumizaka H. Single-stranded DNA catenation mediated by human EVL and a type I topoisomerase. Nucleic Acids Res. 2010, 38, 7579–7586. 10.1093/nar/gkq630. 20639531 PMC2995056

[ref35] Han D.; Pal S.; Liu Y.; Yan H. Folding and cutting DNA into reconfigurable topological nanostructures. Nat. Nanotechnol. 2010, 5, 712–717. 10.1038/nnano.2010.193. 20890274 PMC3071358

[ref36] Peil A.; Zhan P.; Liu N. DNA Origami Catenanes Templated by Gold Nanoparticles. Small 2020, 16, 1905987 10.1002/smll.201905987. 31917513

[ref37] List J.; Falgenhauer E.; Kopperger E.; Pardatscher G.; Simmel F. C. Long-range movement of large mechanically interlocked DNA nanostructures. Nat. Commun. 2016, 7, 12414 10.1038/ncomms12414. 27492061 PMC4980458

[ref38] Xiong Q.; Xie C.; Zhang Z.; Liu L.; Powell J. T.; Shen Q.; Lin C. DNA Origami Post-Processing by CRISPR-Cas12a. Angew Chem Int Ed Engl 2020, 59, 3956–3960. 10.1002/anie.201915555. 31883145 PMC7101258

[ref39] Stark W. M.; Sherratt D. J.; Boocock M. R. Site-specific recombination by Tn3 resolvase: topological changes in the forward and reverse reactions. Cell 1989, 58, 779–790. 10.1016/0092-8674(89)90111-6. 2548736

[ref40] Arnold P. H.; Blake D. G.; Grindley N. D.; Boocock M. R.; Stark W. M. Mutants of Tn3 resolvase which do not require accessory binding sites for recombination activity. EMBO J 1999, 18, 1407–1414. 10.1093/emboj/18.5.1407. 10064606 PMC1171230

[ref41] McIlwraith M. J.; Boocock M. R.; Stark W. M. Site-specific recombination by Tn3 resolvase, photocrosslinked to its supercoiled DNA substrate. J. Mol. Biol. 1996, 260, 299–303. 10.1006/jmbi.1996.0400. 8757793

[ref42] Olorunniji F. J.; He J.; Wenwieser S. V.; Boocock M. R.; Stark W. M. Synapsis and catalysis by activated Tn3 resolvase mutants. Nucleic Acids Res. 2008, 36, 7181–7191. 10.1093/nar/gkn885. 19015124 PMC2602789

[ref43] Stark W. M.; Parker C. N.; Halford S. E.; Boocock M. R. Stereoselectivity of DNA catenane fusion by resolvase. Nature 1994, 368, 76–78. 10.1038/368076a0. 8107889

[ref44] Kilby N. J.; Snaith M. R.; Murray J. A. Site-specific recombinases: tools for genome engineering. Trends Genet. 1993, 9, 413–421. 10.1016/0168-9525(93)90104-P. 8122308

[ref45] Högberg B.; Liedl T.; Shih W. M. Folding DNA origami from a double-stranded source of scaffold. J. Am. Chem. Soc. 2009, 131, 9154–9155. 10.1021/ja902569x. 19566089 PMC2724999

[ref46] Bednarz A. L.; Boocock M. R.; Sherratt D. J. Determinants of correct res site alignment in site-specific recombination by Tn3 resolvase. Genes Dev. 1990, 4, 2366–2375. 10.1101/gad.4.12b.2366. 2177716

[ref47] Douglas S. M.; Marblestone A. H.; Teerapittayanon S.; Vazquez A.; Church G. M.; Shih W. M. Rapid prototyping of 3D DNA-origami shapes with caDNAno. Nucleic Acids Res. 2009, 37, 5001–5006. 10.1093/nar/gkp436. 19531737 PMC2731887

