# Supplementary material for: Topogami: Topologically Linked DNA Origami
Source: ACS Nanosci Au. 2021 Nov 12;2(1):57–63. doi: 10.1021/acsnanoscienceau.1c00027 (PMC8861903; doi:10.1021/acsnanoscienceau.1c00027)
Supplement: Supplementary file 1 — ng1c00027_si_001.pdf [file ng1c00027_si_001.pdf]

Supplementary information

TOPOGAMI: Topologically linked DNA origami

Yusuke Sakai ‡, a, †, Gerrit D. Wilkens ‡, a, b, Karol Wolski c, Szczepan Zapotoczny c, and Jonathan G Heddle a, *

a. Bionanoscience and Biochemistry Laboratory, Malopolska Centre of Biotechnology, Jagiellonian University, Gronostajowa 7A, 30-387 Krakow

b. Postgraduate School of Molecular Medicine, Żwirki I Wigury 61, 02-091 Warsaw

c. Faculty of Chemistry, Jagiellonian University, Gronostajowa 2, 30-387 Krakow

‡. equal contribution

†. Present adress: Laboratory for Integrated Biodevice, Centre for Biosystems Dynamics Research, RIKEN, 1-3 Yamadaoka Suita, Osaka, Japan

Optimisation of plasmid amplification

Initially *E. coli* DH5 alpha was used for the amplification of pMA21. We frequently encountered a single contaminating band in the resulting plasmid preparations (Fig. S1) that produced a linear band of 3 kbp after B*amH*I/*Sca*I double digest. We presumed that the observed band was the resolution product from pMA21 containing the *Sca*I restriction site as well as the origin of replication which is formed due to low levels of recombinase being expressed. Switching to the plasmid amplification strain to DH5 (a gift of M. Stark) as outlined in the materials and methods eliminated this problem.

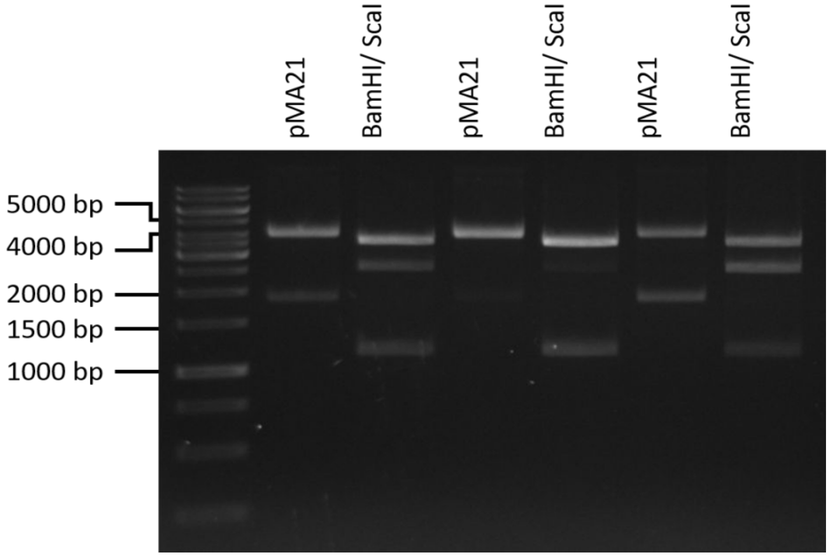

Figure S1: Amplification of pMA21 in *E. coli* Dh5 alpha leads to appearance of a contaminating band running at 3000 bp after restriction digest.

caDNANo diagram of DNA origami design

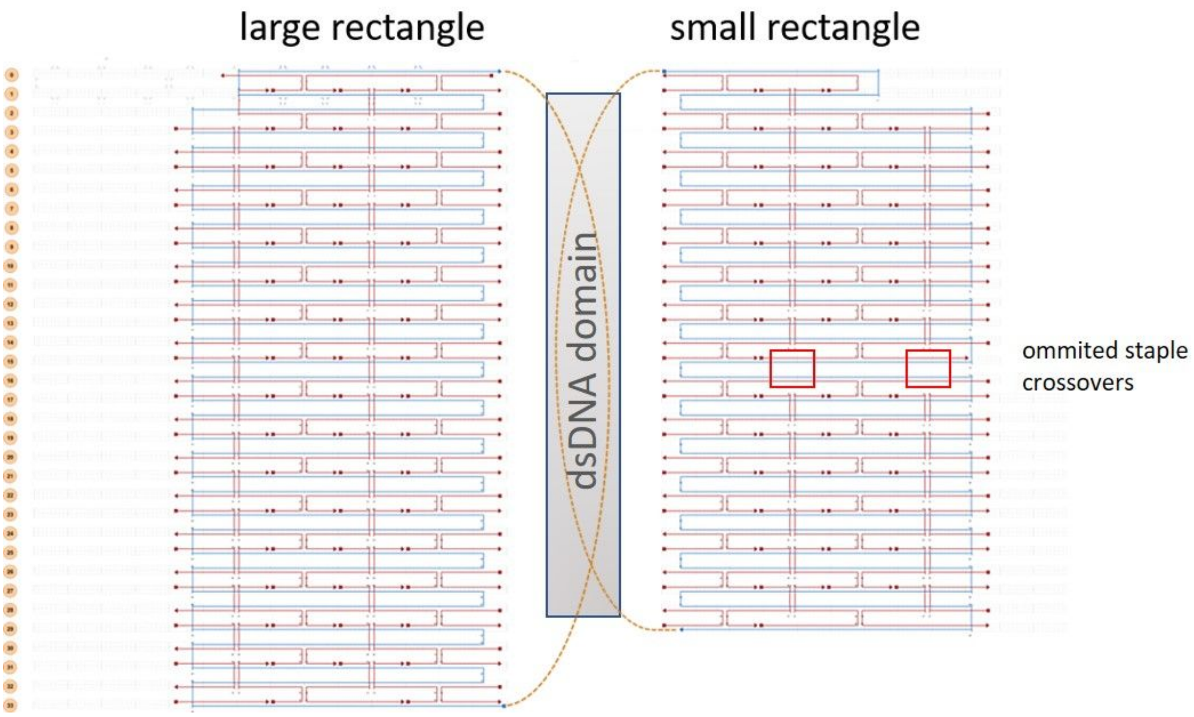

Figure S2: CaDNANo diagram of rectangle catenanes. Blue and red lines indicated scaffold and staple strands respectively. The "open" appearance of the small rectangle was achieved by removing staple crossovers between the 15th and 16th helices (removed crossovers indicated by red rectangles). The scaffold of both structures is interlocked by an outside scaffold loop shown by the orange dotted lines that forms a double helix domain in between both structures.

Scaffold sequences

Large circle sequence:

```
CAACCGTTCGAAATATTATAAATTATCAGACATAGTAAAACGGCTTCGTTTGAGTGTCCATTAAATCGTCATTTT
GGCATAATAGACACATCGTGTCTGATATTCGATTTAAGGTACATTTTTATGCGAATTCTTTATCCGCTTCATCAAG
ATGAAAATAACGCGCCAGCTGCCTCGCGCGTTTCGGTGATGACGGTGAAAACCTCTGACACATGCAGCTCCCG
GAGACGGTCACAGCTTGTCTGTAAGCGGATGCCGGGAGCAGACAAGCCCGTCAGGGCGCGTCAGCGGGTGTT
GGCGGGTGTCGGGGCGCAGCCATGACCCAGTCACGTAGCGATAGCGGAGTGTATACTGGCTTAACTATGCGG
CATCAGAGCAGATTGTACTGAGAGTGCACCATATGCGGTGTGAAATACCGCACAGATGCGTAAGGAGAAAATA
CCGCATCAGGCGCTCTTCCGCTTCCTCGCTCACTGACTCGCTGCGCTCGGTCGTTCGGCTGCGGCGAGCGGTAT
CAGCTCACTCAAAGGCGGTAATACGGTTATCCACAGAATCAGGGGATAACGCAGGAAAGAACATGTGAGCAA
AAGGCCAGCAAAAGGCCAGGAACCGTAAAAAGGCCGCGTTGCTGGCGTTTTTCCATAGGCTCCGCCCCCCTGA
CGAGCATCACAAAAATCGACGCTCAAGTCAGAGGTGGCGAAACCCGACAGGACTATAAAGATACCAGGCGTTT
CCCCCTGGAAGCTCCCTCGTGCGCTCTCCTGTTCCGACCCTGCCGCTTACCGGATACCTGTCCGCCTTTCTCCCTT
CGGGAAGCGTGGCGCTTTCTCATAGCTCACGCTGTAGGTATCTCAGTTCGGTGTAGGTCGTTCGCTCCAAGCTG
GGCTGTGTGCACGAACCCCCCGTTCAGCCCGACCGCTGCGCCTTATCCGGTAACTATCGTCTTGAGTCCAACCC
GGTAAGACACGACTTATCGCCACTGGCAGCAGCCACTGGTAACAGGATTAGCAGAGCGAGGTATGTAGGCGG
TGCTACAGAGTTCTTGAAGTGGTGGCCTAACTACGGCTACACTAGAAGGACAGTATTTGGTATCTGCGCTCTGC
TGAAGCCAGTTACCTTCGGAAAAAGAGTTGGTAGCTCTTGATCCGGCAAACAAACCACCGCTGGTAGCGGTGG
TTTTTTTGTTTGCAAGCAGCAGATTACGCGCAGAAAAAAAGGATCTCAAGAAGATCCTTTGATCTTTTCTACGGG
GTCTGACGCTCAGTGGAACGAAAACTCACGTTAAGGGATTTTGGTCATGAGATTATCAAAAAGGATCTTCACCT
AGATCCTTTTAAATTAAAAATGAAGTTTTAAATCAATCTAAAGTATATATGAGTAAACTTGGTCTGACAGTTACC
AATGCTTAATCAGTGAGGCACCTATCTCAGCGATCTGTCTATTTCGTTCATCCATAGTTGCCTGACTCCCCGTCGT
GTAGATAACTACGATACGGGAGGGCTTACCATCTGGCCCCAGTGCTGCAATGATACCGCGAGACCCACGCTCA
CCGGCTCCAGATTTATCAGCAATAAACCAGCCAGCCGGAAGGGCCGAGCGCAGAAGTGGTCCTGCAACTTTAT
```

CCGCCTCCATCCAGTCTATTAATTGTTGCCGGGAAGCTAGAGTAAGTAGTTCGCCAGTTAATAGTTTGCGCAAC
GTTGTTGCCATTGCTGCAGGCATCGTGGTGTCACGCTCGTCGTTTGGTATGGCTTCATTCAGCTCCGGTTCCCAA
CGATCAAGGCGAGTTACATGATCCCCCATGTTGTGCAAAAAAGCGGTTAGCTCCTTCGGTCCTCCGATCGTTGT
CAGAAGTAAGTTGGCCGCAGTGTTATCACTCATGGTTATGGCAGCACTGCATAATTCTCTTACTGTCATGCCATC
CGTAAGATGCTTTTCTGTGACTGGTGAGTACTCAACCAAGTCATTCTGAGAATAGTGTATGCGGCGACCGAGTT
GCTCTTGCCCGGCGTCAACACGGGATAATACCGCGCCACATAGCAGAACTTTAAAAGTGCTCATCATTGGAAAA
CGTTCTTCGGGGCGAAAACTCTCAAGGATCTTACCGCTGTTGAGATCCAGTTCGATGTAACCCACTCGTGCACCC
AACTGATCTTCAGCATCTTTTACTTTCACCAGCGTTTCTGGGTGAGCAAAAACAGGAAGGCAAAATGCCGCAAA
AAAGGGAATAAGGGCGACACGGAAATGTTGAATACTCATACTCTTCCTTTTTCAATATTATTGAAGCATTTATCA
GGGTTATTGTCTCATGAGCGGATACATATTTGAATGTATTTAGAAAAATAAACAAATAGGGGTTCCGCGCACAT
TTCCCCGAAAAGTGCCACCTGACGTCTAAGAAACCATTATTATCATGACATTAACCTATAAAAATAGGCGTATCA
CGAGGCCCTTTCGTCTTCAAGAATTCTTTATCCGCTTCATCAAGATGAAAATAACGCGCCAGCTGAAGTTCATCG
GGTTCGCCAGTGAACCTGCCATAACTCTCAACCTGCTCAGTGGTCAAAAAATCAACGGGCATATCGGCCTCCCT
GCCTGACGGCTTTTTTAACACAACTG

Small circle sequence:

CAGTTGTGTTAAAAAAGCCGTCAGGCAGGGAGGCCGATATGCCCGTTGATTTTTTGACCACTGAGCAGGTTGA
GAGTTATGGCAGGTTCACTGGCGAACCCGATGAACTTCAGCTGCGGTAAAGCTCATCAGCGTGGTCGTGAAGC
GATTCACAGATGTCTGCCTGTTCATCCGCGTCCAGCTCGTTGAGTTTCTCCAGAAGCGTTAATGTCTGGCTTCTG
ATAAAGCGGGCCATGTTAAGGGCGGTTTTTTCCTGTTTGGTCACTGATGCCTCCGTGTAAGGGGGATTTCTGTT
CATGGGGGTAATGATACCGATGAAACGAGAGAGGATGCTCACGATACGGGTTACTGATGATGAACATGCCCG
GTTACTGGAACGTTGTGAGGGTAAACAACTGGCGGTATGGATGCGGCGGGACCAGAGAAAAATCACTCAGGG
TCAATGCCAGCGCTTCGTTAATACAGATGTAGGTGTTCCACAGGGTAGCCAGCAGCATCCTGCGATGCAGATCC
GGAACATAATGGTGCAGGGCGCTGACTTCCGCGTTTCCAGACTTTACGAAACACGGAAACCGAAGACCATTCA
TGTTGTTGCTCAGGTCGCAGACGTTTTGCAGCAGCAGTCGCTTCACGTTCGCTCGCGTATCGGTGATTCATTCTG
CTAACCAGTAAGGCAACCCCGCCAGCCTAGCCGGGTCCTCAACGACAGGAGCACGATCATGCGCACCCGTGGC
CAGGACCCAACGCTGCCCGAGATGCGCCGCGTGCGGCTGCTGGAGATGGCGGACGCGATGGATATGTTCTGC
CAAGGGTTGGTTTGCGCATTCACAGTTCTCCGCAAGAATTGATTGGCTCCAATTCTTGGAGTGGTGAATCCGTT
AGCGAGGTGCCGCCGGCTTCCATTCAGGTCGAGGTGGCCCGGCTCCATGCACCGCGACGCAACGCGGGGAGG
CAGACAAGGTATAGGGCGGCGCCTACAATCCATGCCAACCCGTTCCATGTGCTCGCCGAGGCGGCATAAATCG
CCGTGACGATCAGCGGTCCAGTGATCGAAGTTAGGCTGGTAAGAGCCGCGAGCGATCCTTGAAGCTGTCCCTG
ATGGTCGTCATCTACCTGCCTGGACAGCATGGCCTGCAACGCGGGCATCCCGATGCCGCCGGAAGCGAGAAGA
ATCATAATGGGGAAGGCCATCCAGCCTCGCGTCGCGAACGCCAGCAAGACGTAGCCCAGCGCGTCGGCCGCCA
TGCCGGCGATAATGGCCTGCTTCTCGCCGAAACGTTTGGTGGCGGGACCAGTGACGAAGGCTTGAGCGAGGG
CGTGCAAGATTCCGAATACCGCAAGCGACAGGCCGATCATCGTCGCGCTCCAGCGAAAGCGGTCCTCGCCGAA
AATGACCCAGAGCGCTGCCGGCACCTGTCCTACGAGTTGCATGATAAAGAAGACAGTCATAAGTGCGGCGACG
ATAGTCATGCCCCGCGCCCACCGGAAGGAGCTGACTGGGTTGAAGGCTCTCAAGGGCATCGGTCGACGCTCTC
CCTTATGCGACTCCTGCATTAGGAAGCAGCCCAGTAGTAGGTTGAGGCCGTTGAGCACCGCCGCCGCAAGGAA
TGGTGCATGCAAGGAGATGGCGCCCAACAGTCCCCCGGCCACGGGGCCTGCCACCATACCCACGCCGAAACAA
GCGCTCATGAGCCCGAAGTGGCGAGCCCGATCTTCCCCATCGGTGATGTCGGCGATATAGGCGCCAGCAACCG
CACCTGTGGCGCCGGTGATGCCGGCCACGATGCGTCCGGCGTAGAGGATCCACAGGACGGGTGTGGTCGCCA
TGATCGCGTAGTCGATAGTGGCTCCAAGTAGCGAAGCGAGCAGGACTGGGCGGCGGCCAAAGCGGTCGGACA
GTGCTCCGAGAACGGGTGCGCATAGAAATTGCATCAACGCATATAGCGCTAGCAGCACGCCATAGTGACTGGC
GATGCTGTCGGAATGGACGATATCCCGCAAGAGGCCCGGCAGTACCGGCATAACCAAGCCTATGCCTACAGCA
TCCAGGGTGACGGTGCCGAGGATGACGATGAGCGCATTGTTAGATTTCATACACGGTGCCTGACTGCGTTAGC
AATTTAACTGTGATAAACTACCGCATTAAAGCTTATCGATGATAAGCTGTCAAAGCTCTTCATGAGAATTCGCAT
AAAAATGTACCTTAAATCGAATATCAGACACGATGTGTCTATTATGCCAAAATGACGATTTAATGGACACTCAA
ACGAAGCCGTTTTACTATGTCTGATAATTTATAATATTTCGAACGGTTG

Staple list for large rectangle

001 0H108-1H110 Large	TATCCGCTTCATCGTAAGCGGATGTTTT
002 1H56-0H39 Large	TCTGACACGTTTCGGTGATGACGGTTTT
003 1H72-3H71 Large	CCCGGAGACGTCAGCGGGTGTTGGTGGCTTAA
004 1H88-1H71 Large	GCTTGTCTAAGATGAAAATAACGCGCCAGCTGCCTCGCGCATGCAGCT
005 2H110-3H110 Large	TTTTCCGGGAGCAGAACTGAGAGTGCTTTT
006 3H33-4H33 Large	TTTTGTCACGTAGCGTCTTCCGCTTCTTTT
007 3H56-1H55 Large	GTGTATACCGGGTGTCGGGGCGCATGAAAACC
008 3H72-5H71 Large	CTATGCGGGATGCGTAAGGAGAAACGTTCGGC
009 3H88-1H87 Large	CAGATTGTCAAGCCCGTCAGGGCGCGGTCACA
010 4H110-5H110 Large	TTTTACCATATGCGGCAAAGGCGGTATTTT
011 5H33-6H33 Large	TTTTCTCGCTCACTGGCAAAAGGCCATTTT
012 5H56-3H55 Large	CGCTCGGTATACCGCATCAGGCGCATAGCGGA
013 5H72-7H71 Large	TGCGGCGAAACGCAGGAAAGAACACGTTTTTC
014 5H88-3H87 Large	AGCTCACTTGTGAAATACCGCACACATCAGAG
015 6H110-7H110 Large	TTTTATACGGTTATCCATCACAAAAATTTT
017 7H56-5H55 Large	GTTGCTGGTGTGAGCAAAAGGCCAACTCGCTG
019 7H88-5H87 Large	CTGACGAGCACAGAATCAGGGGATGCGGTATC
050 20H110-21H110 Large	TTTTCAGTGCTGCAATTGTTGCCGGGTTTT
051 21H33-22H33 Large	TTTTGGAAGGGCCGAGGCATCGTGGTTTTT
053 21H72-23H71 Large	ATCCGCCTATAGTTTGCGCAACGTATTCAGCT
055 22H110-23H110 Large	TTTTAAGCTAGAGTATTACATGATCCTTTT
056 23H33-24H33 Large	TTTTGTCACGCTCGTGTTGGCCGCAGTTTT
057 23H56-21H55 Large	ATGGCTTCTGTTGCCATTGCTGCAGCGCAGAA
058 23H72-25H71 Large	CCGGTTCCTCCTTCGGTCCTCCGATGCATAAT
059 23H88-21H87 Large	AAGGCGAGAGTAGTTCGCCAGTTACCATCCAG

060 24H110-25H110 Large	TTTTCCCATGTTGTGAGATGCTTTTCTTTT
061 25H33-26H33 Large	TTTTTGTTATCACTCGCTCTTGCCCGTTTT
062 25H56-23H55 Large	GGCAGCACTCGTTGTCAGAAGTAACGTTTGGT
063 25H72-27H71 Large	TCTCTTACATTCTGAGAATAGTGTCATAGCAG
064 25H88-23H87 Large	CATCCGTACAAAAAAGCGGTTAGCCAACGATC
065 26H110-27H110 Large	TTTTTGTGACTGGTGAAAACGTTCTTTTTT
066 27H33-28H33 Large	TTTTGCGTCAACACGCGTGCACCCAATTTT
067 27H56-25H55 Large	CCGCGCCAATGCGGCGACCGAGTTATGGTTAT
068 27H72-29H71 Large	AACTTTAACGCTGTTGAGATCCAGCAGCGTTT
069 27H88-25H87 Large	ATCATTGGAGTACTCAACCAAGTCTGTCATGC
070 28H110-29H110 Large	TTTTCGGGGCGAAAAAAAATGCCGCATTTT
071 29H33-30H33 Large	TTTTCTGATCTTCAGTTATTGAAGCATTTT
072 29H56-27H55 Large	ACTTTCACTTCGATGTAACCCACTGGATAATA
073 29H72-31H71 Large	CTGGGTGATGTTGAATACTCATACGATACATA
074 29H88-27H87 Large	AGGAAGGCCTCTCAAGGATCTTACAAGTGCTC
075 30H110-31H110 Large	TTTTAAAAAGGGAATCAAATAGGGGTTTTT
076 31H33-32H33 Large	TTTTTTTATCAGGGTCATTAACCTATTTTT
077 31H56-29H55 Large	CATGAGCGTCTTCCTTTTTCAATACATCTTTT
078 31H72-33H87 Large	TTTGAATGACCTGACGTCTAAGAACGTCTTCAAGAATTCTTTATCCGC
079 31H88-29H87 Large	AAAATAAAAAGGGCGACACGGAAAGCAAAAAC
080 32H110-33H110 Large	TTTTTCCGCGCACATGATGAAAATAACGCG
081 33H33-33H55 Large	TTTTAAAAATAGGCGTATCACGA
082 33H56-31H55 Large	GGCCCTTTACCATTATTATCATGATATTGTCT
083 33H88-31H87 Large	TTCATCAATTCCCCGAAAAGTGCCTATTTAGA

Staple list for small rectangle

084 1H145-0H145 Small	TTTTCGCATTGTTAGAGCTGTCAAACATGA
085 1H168-3H167 Small	CACGGTGCGGTGACGGTGCCGAGGTAGCAGCA
086 1H184-1H167 Small	GTTAGCAAATAAACTACCGCATTAAAGCTTATCGATGATAATTTCATA

087 2H222-3H199 Small	TTTTCCGGCAGTACCGGCATAACCAAGCCTACGGAATGG
088 3H145-2H145 Small	TTTTGCATATAGCGCATGACGATGAGTTTT
089 3H168-5H167 Small	CGCCATAGACGGGTGCGCATAGAAAGGACGGG
090 3H184-1H183 Small	GATGCTGTTGCCTACAGCATCCAGCTGACTGC
091 3H200-5H199 Small	ACGATATCGGCGGCCAAAGCGGTCGATAGTGG
092 4H222-3H222 Small	TTTTCAGGACTGGGCCCGCAAGAGGCTTTT
093 5H145-4H145 Small	TTTTAGAGGATCCACATTGCATCAACTTTT
094 5H168-7H167 Small	TGTGGTCGTGATGCCGGCCACGATCCACGCCG
095 5H184-3H183 Small	GCGTAGTCGGACAGTGCTCCGAGATGACTGGC
096 5H200-7H199 Small	CTCCAAGTATAGGCGCCAGCAACCTGGCGAGC
097 6H222-5H222 Small	TTTTATGTCGGCGATAGCGAAGCGAGTTTT
098 7H145-6H145 Small	TTTTTGCCACCATACGCGTCCGGCGTTTTT
099 7H168-9H167 Small	AAACAAGCGCCCAACAGTCCCCCGACGCTCTC
100 7H184-5H183 Small	GCCCGAAGGCACCTGTGGCGCCGGCCATGATC
101 7H200-9H199 Small	CCGATCTTGCCGCAAGGAATGGTGAGCAGCCC
102 8H222-7H222 Small	TTTTTGAGCACCGCCCCCCATCGGTGTTTT
103 9H145-8H145 Small	TTTTGGCATCGGTCGGCCACGGGGCCTTTT
104 9H168-11H167 Small	CCTTATGCGAGCTGACTGGGTTGAAAAATGAC
105 9H184-7H183 Small	CATTAGGACATGCAAGGAGATGGCGCTCATGA
106 9H200-11H199 Small	AGTAGTAGCGACGATAGTCATGCCTACGAGTT
107 10H222-9H222 Small	TTTTCATAAGTGCGGGTTGAGGCCGTTTTT
108 11H145-10H145 Small	TTTTGGTCCTCGCCGAGGCTCTCAAGTTTT
109 11H168-13H167 Small	CCAGAGCGCGATCATCGTCGCGCTCGGCGATA
110 11H184-9H183 Small	ACCTGTCCCCGCGCCCACCGGAAGGACTCCTG
111 11H200-13H199 Small	GCATGATAGTGCAAGATTCCGAATTTGGTGGC
112 12H222-11H222 Small	TTTTTGAGCGAGGGCAAGAAGACAGTTTTT
113 13H145-12H145 Small	TTTTGGCCGCCATGCCCAGCGAAAGCTTTT
114 13H168-15H167 Small	ATGGCCTGCGCCAGCAAGACGTAGATCTACCT
115 13H184-11H183 Small	CGAAACGTACCGCAAGCGACAGGCCTGCCGGC

116 13H200-15H199 Small	GGGACCAGATGGGGAAGGCCATCCCGGGCATC
117 14H222-13H222 Small	TTTTGAAGAATCATATGACGAAGGCTTTTT
118 15H145-14H145 Small	TTTTTGATGGTCGTCCCCAGCGCGTCTTTT
119 15H168-17H167 Small	GCCTGGACGCCGCGAGCGATCCTTGCGGCGCC
120 15H184-13H183 Small	CTGCAACGAGCCTCGCGTCGCGAACTTCTCGC
122 16H222-15H222 Small	TTTTCGTGACGATCAGCCGGAAGCGATTTT
123 17H145-16H145 Small	TTTTCAAGGTATAGGGAAGCTGTCCCTTTT
124 17H168-19H167 Small	TACAATCCCACCGCGACGCAACGCACAGTTCT
125 17H184-15H183 Small	CCGTTCCAAGTTAGGCTGGTAAGAAGCATGGC
126 17H200-19H199 Small	CCGAGGCGTTCCATTCAGGTCGAGTCTTGGAG
127 18H222-17H222 Small	TTTTGTGCCGCCGGCGCATAAATCGCTTTT
128 19H145-18H145 Small	TTTTTTTGCGCATTCGGGGAGGCAGATTTT
129 19H168-21H167 Small	CCGCAAGACGATGGATATGTTCTGCTAGCCGG
130 19H184-17H183 Small	GCTCCAATGTGGCCCGGCTCCATGATGCCAAC
131 19H200-21H199 Small	TGGTGAATGCGCCGCGTGCGGCTGATGCGCAC
132 20H222-19H222 Small	TTTTCTGCCCGAGATCCGTTAGCGAGTTTT
133 21H145-20H145 Small	TTTTACCCCGCCAGCCCAAGGGTTGGTTTT
134 21H168-23H167 Small	GTCCTCAATGATTCATTCTGCTAATTCCAGAC
135 21H184-19H183 Small	GCACGATCCTGGAGATGGCGGACGATTGATTG
136 21H200-23H199 Small	CCGTGGCCCAGCAGTCGCTTCACGCCATTCAT
137 22H222-21H222 Small	TTTTACGTTTTGCAGAGGACCCAACGTTTT
138 23H145-22H145 Small	TTTTGACTTCCGCGTCCAGTAAGGCATTTT
139 23H168-25H167 Small	TTTACGAAATCCGGAACATAATGGATGCGGCG
140 23H184-21H183 Small	ACCGAAGATTCGCTCGCGTATCGGCGACAGGA
141 23H200-25H199 Small	GTTGTTGCCACAGGGTAGCCAGCATCAATGCC
142 24H222-23H222 Small	TTTTTGTAGGTGTTCTCAGGTCGCAGTTTT
143 25H145-24H145 Small	TTTTTGGCGGTATGGTGCAGGGCGCTTTTT
144 25H168-27H167 Small	GGACCAGATACTGGAACGTTGTGAATGCCTCC
145 25H184-23H183 Small	ACTCAGGGGCATCCTGCGATGCAGACACGGAA

146 25H200-27H199 Small	AGCGCTTCGATACGGGTTACTGATGGGGTAAT
147 26H222-25H222 Small	TTTTAGGATGCTCACGTTAATACAGATTTT
148 27H145-26H145 Small	TTTTTTTGGTCACTGGGGTAAACAACTTTT
149 27H168-29H167 Small	GTGTAAGGGGCCATGTTAAGGGCGATCAGCGT
150 27H184-25H183 Small	TGTTCATGGATGAACATGCCCGGTGAAAAATC
151 27H200-29H199 Small	GATACCGAAGAAGCGTTAATGTCTCTGCCTGT
152 28H222-27H222 Small	TTTTTGAGTTTCTCCTGAAACGAGAGTTTT
153 29H145-28H145 Small	TTTTCGGTAAAGCTCGTTTTTTCCTGTTTT
154 29H168-27H183 Small	GGTCGTGAAGCGATTCACAGATGTGGCTTCTGATAAAGCGGGGATTTC
155 29H200-29H222 Small	TCATCCGCGTCCAGCTCGTTTTT

Statistical analysis of AFM images

Figure S3: AFM image of 184 topogami particles. #1 (left) and #2 (right). White: Unclear or broken structures; Orange: Obviously paired rectangles; Blue: Isolated rectangles.

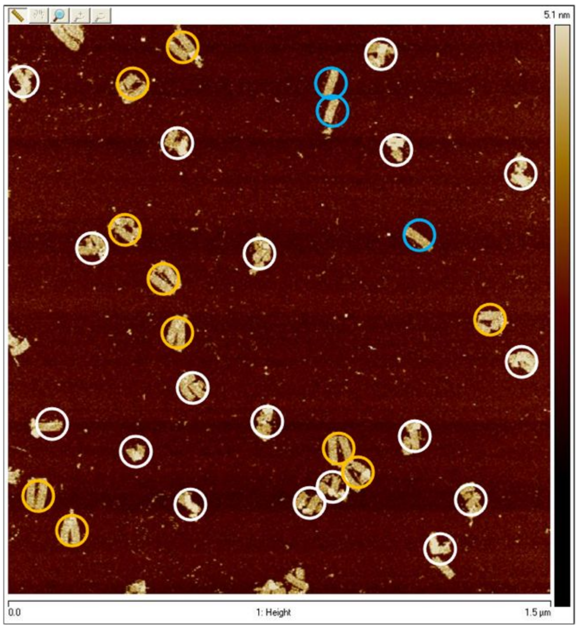

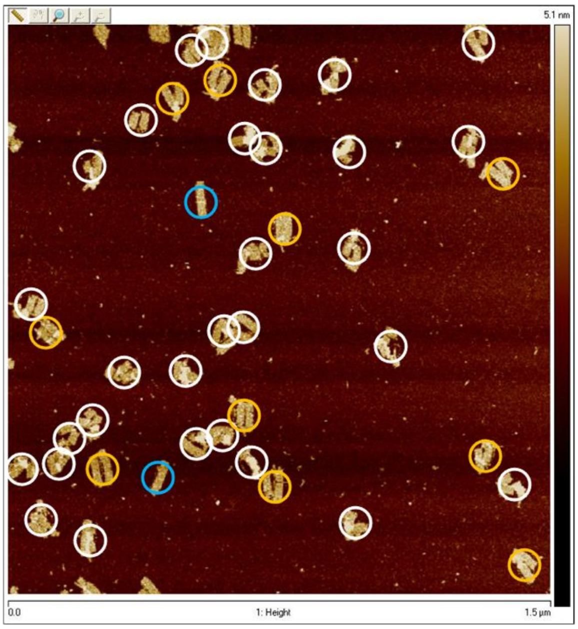

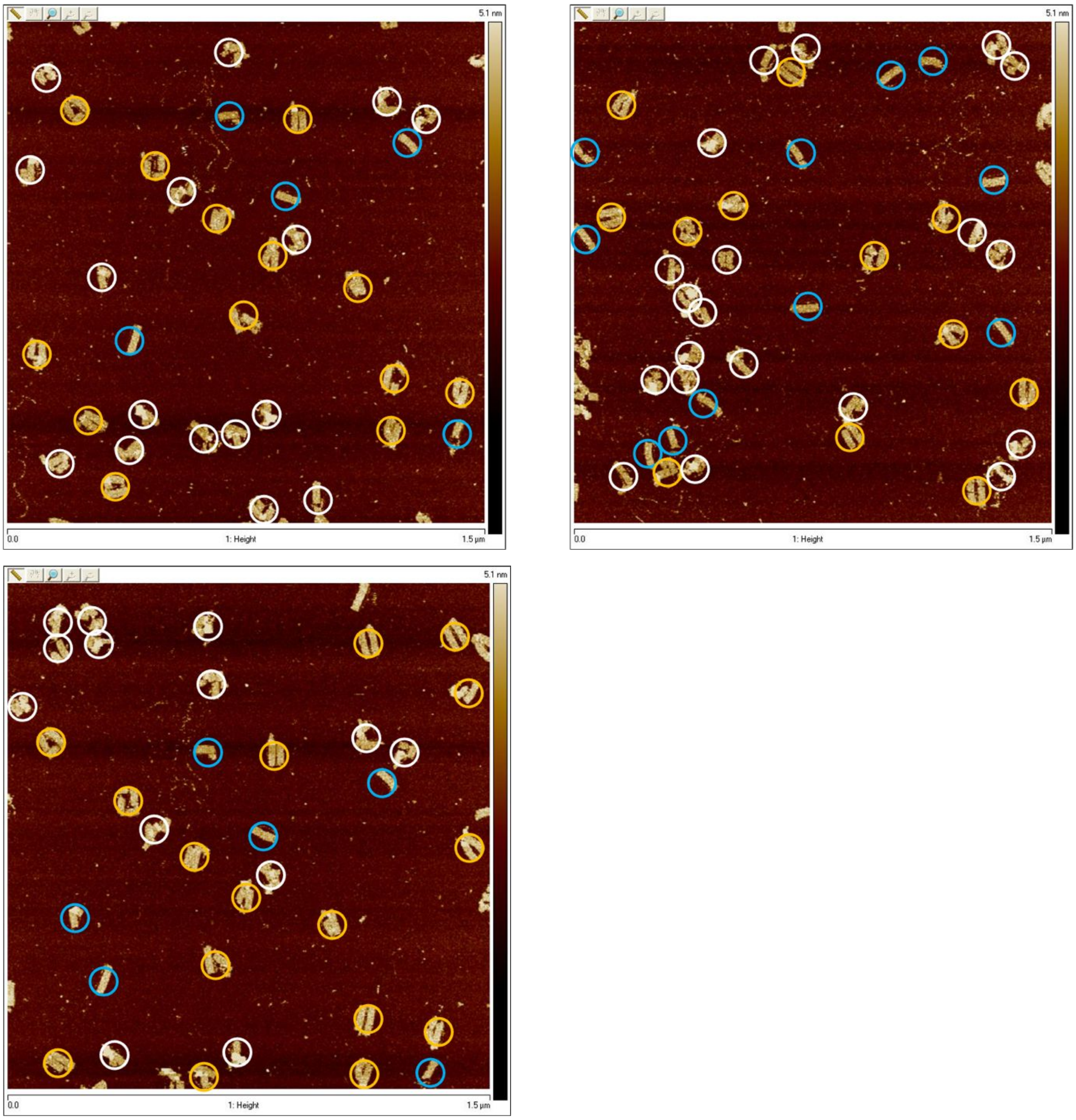

Figure S3 (Subsequent): AFM image of 184 topogami particles. #3 (left top), #4 (right top) and #5 (left bottom). White: Unclear or broken structures; Orange: Obviously paired rectangles; Blue: Isolated rectangles.

Table S1: Summary of AFM image analysis of 5 datasets.

							**Including broken structures**		**Excluding broken structures**	
**Class**	**#1**	**#2**	**#3**	**#4**	**#5**	**Total**	**Frequency**	**Error**	**Frequency**	**Error**
Paired rectangle (orange)	13	12	10	10	16	61	**33 %**	3.5 %	69 %	4.9 %
Isolated rectangle (blue)	5	11	3	2	6	27	**15 %**	2.6 %	31 %	4.9 %
Unclear/Broken (white)	16	20	18	30	12	96	52 %	3.7 %	-	-
Total	34	43	31	42	34	184				

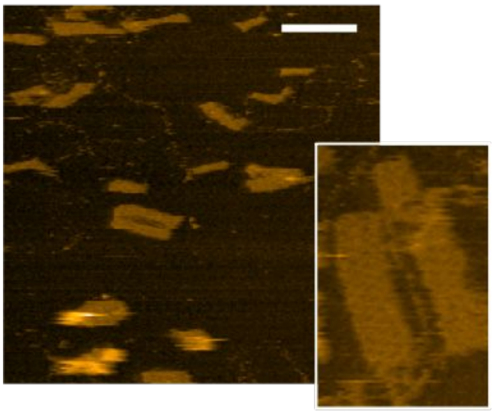

Figure S4: High speed AFM image of topogami. HS-AFM imaging enabled fine and high-resolution observation of topogami structure. Along with horizontal staple crossover pattern on rectangular bodies, double stranded scaffold loop sticking out from corners of each body was observed. Scale bar is 100 nm. Right inset is 128 nm×84 nm square magnified image.

Additional DNA topogami T-shape structure

Here an additional “T” shape structure is shown that is paired with a rectangle that was used in the previous design. Note, as this is a proof of principle, we did not optimise assembly conditions for this structure.

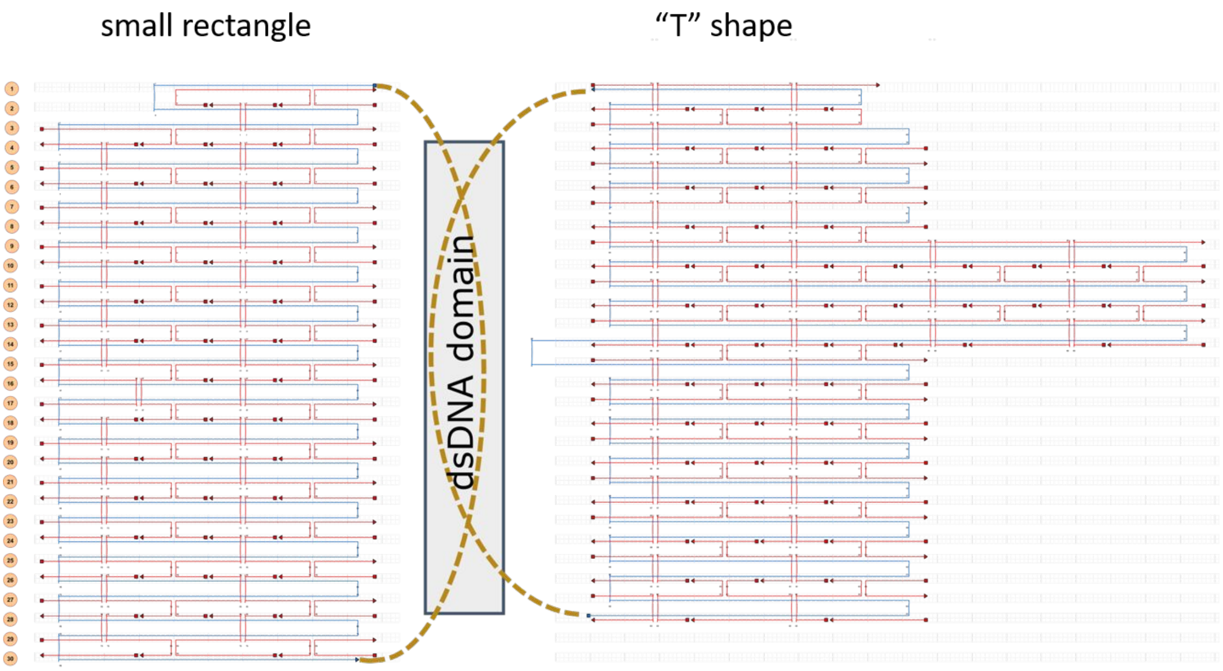

Figure S5: CaDNANo diagram of topogami rectangle and "T" shape catenanes. Blue and red lines indicate scaffold and staple strands respectively. The scaffold of both structures is interlocked by an outside scaffold loop shown by the orange dotted lines that forms a double helix domain in between both structures.

Staple list for "T" shape DNA origami.

003 1H72-3H71 Large	CCCGGAGACGTCAGCGGGTGTTGGTGGCTTAA
004 1H88-1H71 Large	GCTTGTCTAAGATGAAAATAACGCGCCAGCTGCCTCGCGCATGCAGCT
005 2H110-3H110 Large	TTTTCCGGGAGCAGAACTGAGAGTGCTTTT
006 3H33-4H33 Large	TTTTGTCACGTAGCGTCTTCCGCTTCTTTT
007 3H56-1H55 Large	GTGTATACCGGGTGTCGGGGCGCATGAAAACC
008 3H72-5H71 Large	CTATGCGGGATGCGTAAGGAGAAACGTTCGGC
009 3H88-1H87 Large	CAGATTGTCAAGCCCGTCAGGGCGCGGTCACA
010 4H110-5H110 Large	TTTTACCATATGCGGCAAAGGCGGTATTTT
011 5H33-6H33 Large	TTTTCTCGCTCACTGGCAAAAGGCCATTTT
012 5H56-3H55 Large	CGCTCGGTATACCGCATCAGGCGCATAGCGGA
013 5H72-7H71 Large	TGCGGCGAAACGCAGGAAAGAACACGTTTTTC
014 5H88-3H87 Large	AGCTCACTTGTGAAATACCGCACACATCAGAG
015 6H110-7H110 Large	TTTTATACGGTTATCCATCACAAAAATTTT

017 7H56-5H55 Large	GTTGCTGGTGTGAGCAAAAGGCCAACTCGCTG
019 7H88-5H87 Large	CTGACGAGCACAGAATCAGGGGATGCGGTATC
050 20H110-21H110 Large	TTTTCAGTGCTGCAATTGTTGCCGGGTTTT
051 21H33-22H33 Large	TTTTGGAAGGGCCGAGGCATCGTGGTTTTT
053 21H72-23H71 Large	ATCCGCCTATAGTTTGCGCAACGTATTCAGCT
055 22H110-23H110 Large	TTTTAAGCTAGAGTATTACATGATCCTTTT
056 23H33-24H33 Large	TTTTGTCACGCTCGTGTTGGCCGCAGTTTT
057 23H56-21H55 Large	ATGGCTTCTGTTGCCATTGCTGCAGCGCAGAA
058 23H72-25H71 Large	CCGGTTCCTCCTTCGGTCCTCCGATGCATAAT
059 23H88-21H87 Large	AAGGCGAGAGTAGTTCGCCAGTTACCATCCAG
060 24H110-25H110 Large	TTTTCCCATGTTGTGAGATGCTTTTCTTTT
061 25H33-26H33 Large	TTTTTGTTATCACTCGCTCTTGCCCGTTTT
062 25H56-23H55 Large	GGCAGCACTCGTTGTCAGAAGTAACGTTTGGT
063 25H72-27H71 Large	TCTCTTACATTCTGAGAATAGTGTCATAGCAG
064 25H88-23H87 Large	CATCCGTACAAAAAAGCGGTTAGCCAACGATC
065 26H110-27H110 Large	TTTTTGTGACTGGTGAAAACGTTCTTTTTT
066 27H33-28H33 Large	TTTTGCGTCAACACGCGTGCACCCAATTTT
067 27H56-25H55 Large	CCGCGCCAATGCGGCGACCGAGTTATGGTTAT
068 27H72-29H71 Large	AACTTTAACGCTGTTGAGATCCAGCAGCGTTT
069 27H88-25H87 Large	ATCATTGGAGTACTCAACCAAGTCTGTCATGC
070 28H110-29H110 Large	TTTTCGGGGCGAAAAAAAATGCCGCATTTT
071 29H33-30H33 Large	TTTTCTGATCTTCAGTTATTGAAGCATTTT
072 29H56-27H55 Large	ACTTTCACTTCGATGTAACCCACTGGATAATA
073 29H72-31H71 Large	CTGGGTGATGTTGAATACTCATACGATACATA
074 29H88-27H87 Large	AGGAAGGCCTCTCAAGGATCTTACAAGTGCTC
075 30H110-31H110 Large	TTTTAAAAAGGGAATCAAATAGGGGTTTTT
076 31H33-32H33 Large	TTTTTTTATCAGGGTCATTAACCTATTTTT
077 31H56-29H55 Large	CATGAGCGTCTTCCTTTTTCAATACATCTTTT
078 31H72-33H87 Large	TTTGAATGACCTGACGTCTAAGAACGTCTTCAAGAATTCTTTATCCGC

079 31H88-29H87 Large	AAAATAAAAAGGGCGACACGGAAAGCAAAAAC
080 32H110-33H110 Large	TTTTTCCGCGCACATGATGAAAATAACGCG
081 33H33-33H55 Large	TTTTAAAAATAGGCGTATCACGA
082 33H56-31H55 Large	GGCCCTTTACCATTATTATCATGATATTGTCT
083 33H88-31H87 Large	TTCATCAATTCCCCGAAAAGTGCCTATTTAGA
156 37H161-38H161 T-Large	TTTATCCGCTTCATCGTAAGCGGATGTTTT
157 38H215-37H227 T-Large	TCTGACACGTTTCGGTGATGACGGTTTT
158 44H238-46H232 T-Large	TTTTGGAACCGTAAACCCCTGGAAGCTCCCTGTTCGCTC
159 45H161-46H161 T-Large	TTTTTCGACGCTCAAGTAACTATCGTTTTT
160 46H215-44H216 T-Large	CACGAACCAGATACCAGGCGTTTCAAGGCCGC
161 46H231-48H232 T-Large	CAAGCTGGGATTAGCAGAGCGAGGCTCTTGA
162 46H247-46H264 T-Large	TGTAGGTCCGTGCGCTCTCCTGTTCCGACCCTGCCGCTTACAC GCTGT
163 46H263-48H264 T-Large	AGGTATCTCAGAGTTCTTGAAGTGGAAGCCAG
164 46H279-45H302 T-Large	TCATAGCTCCGGATACCTGTCCGCCTTTCTCCCTTTTTT
165 46H302-47H302 T-Large	TTTTCGGGAAGCGTGTACACTAGAAGTTTT
166 47H161-48H161 T-Large	TTTTCTTGAGTCCAATACGCGCAGAATTTT
167 48H215-46H216 T-Large	CCGCTGGTCAGCCACTGGTAACAGGCTGTGTG
168 48H247-46H248 T-Large	AGTTGGTATATGTAGGCGGTGCTACAGTTCGG
169 48H263-50H248 T-Large	TTACCTTCATTATCAAAAAGGATCTATGAGTAAACTTGGTCTG ACAGT
170 48H279-46H280 T-Large	GCTCTGCTGTGGCCTAACTACGGCGCGCTTTC
171 48H302-49H302 T-Large	TTTTGACAGTATTTGTAAATTAAAAATTTT
172 49H161-50H161 T-Large	TTTTAAAAAGGATCTCCCGTCGTGTATTTT
173 50H183-48H184 T-Large	CCTGACTCCAAGAAGATCCTTTGATTTGCAAG
174 50H215-48H216 T-Large	AGCGATCTTGACGCTCAGTGGAACACAAACCA
175 50H247-48H248 T-Large	TACCAATGGGATTTTGGTCATGAGGGAAAAAG
176 50H279-48H280 T-Large	AAAGTATATTCACCTAGATCCTTTGTATCTGC
177 50H302-50H280 T-Large	TTTTTGAAGTTTTAAATCAATCT
178 51H161-52H161 T-Large	TTTTCAGTGCTGCAATTGTTGCCGGGTTTT
217 44H199-46H200 T-Large	CATAGGCTCCCGACAGGACTATAACCCCGTTC

218 46H183-44H184 T-Large	CTTATCCGGTCAGAGGTGGCGAAACCGCCCCC
219 46H199-48H200 T-Large	AGCCCGACTTATCGCCACTGGCAGAGCGGTGG
220 48H183-46H184 T-Large	CAGCAGATCCCGGTAAGACACGACCGCTGCGC
179 48H199-50H184 T-Large Split	TTTTTTTGTCTTTTCTACGGGGTCGTCTATTTCGTTCATCCATAG TTG
180 48H231-50H216 T-Large Split	TCCGGCAAGAAAACTCACGTTAAGCTTAATCAGTGAGGCACCT ATCTC
181 52H183-52H200 T-Large Split	TCTATTAATGATACCGCGAGACCCACGCTCACCGGCTCCAGCA ACTTT
182 52H215-51H238 T-Large Split	GTGGTCCTGATTTATCAGCAATAAACCAGCCAGCCTTTT

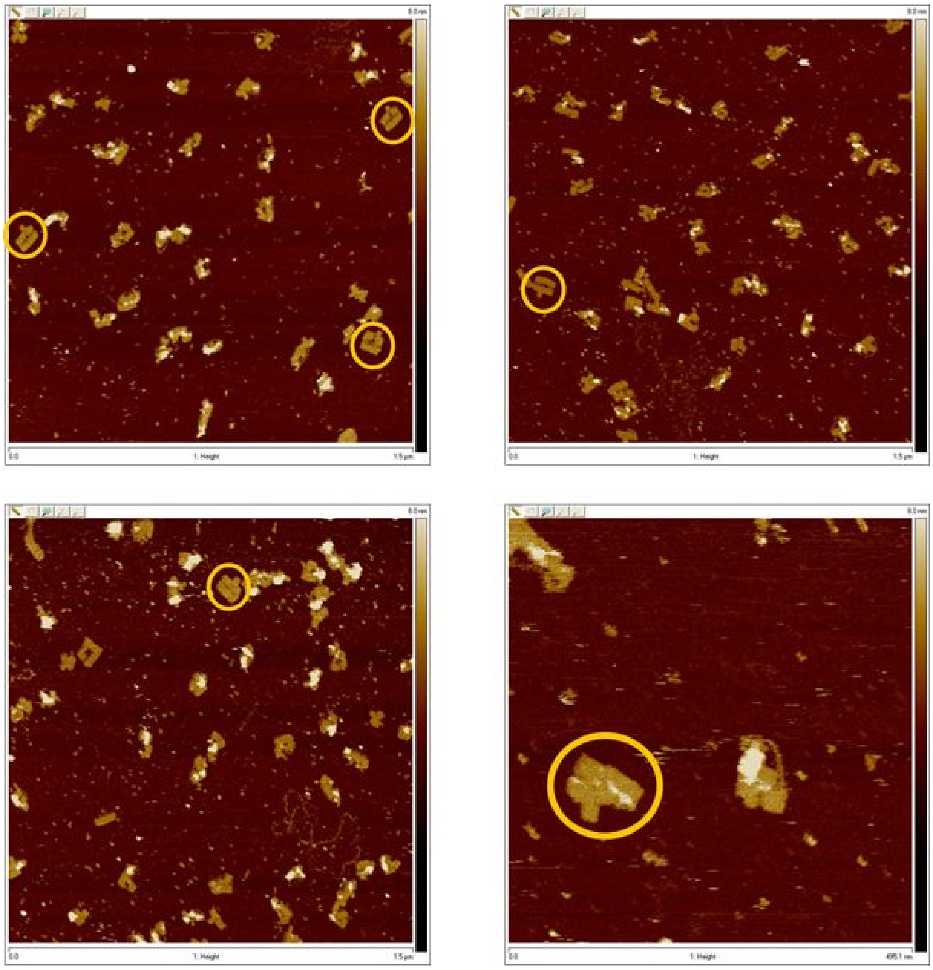

Figure S6. AFM images of DNA topogami catenane consisting of a “T” shaped and a rectangular DNA origami. Orange: paired and folded DNA topogami structures
